# Reclaiming human clinical reasoning in generative AI-enabled healthcare: a perspective on cognitive agency, skill evolution, and accountability

**DOI:** 10.3389/fpubh.2026.1979099

**Published:** 2026-10-13

**Authors:** Jia Xu, Mengjiao Zhang, Bing Zheng, Wu Deng, Rui Shi

**Affiliations:** Department of IT and Data Management of West China Hospital, Sichuan University, Chengdu, China

**Keywords:** clinical decision support systems, clinical reasoning, digital health governance, generative artificial intelligence, human – AI interaction, human-centered AI, narrative cognitive substitution

## Abstract

Generative artificial intelligence (GenAI) and large language models (LLMs) are transforming clinical decision support systems (CDSS) from traditional discrete predictions into fluent, context-aware clinical narrative generation. Although these capabilities substantially improve the efficiency of clinical workflows, they also introduce distinctive cognitive challenges: highly fluent and seemingly plausible medical narratives may reshape the way physicians construct clinical meaning and make analytic judgments. This perspective paper introduces the concept of Narrative Cognitive Substitution (NCS), proposed as a testable mid-level theoretical construct, which describes the hypothesized shift in human cognition from active hypothesis generation to passive retrospective validation when GenAI outputs pre-organize diagnostic reasoning and therapeutic pathways. We further propose a Skill Evolution Framework that characterizes the potential effects of GenAI on physician competence across five trajectories: Augmentation, Restructuring, Deskilling, Never-skilling, and Mis-skilling. In addition, we analyze the resulting Accountability Gap: although algorithmic outputs are increasingly embedded in clinical decision-making, decision influence is distributed across clinicians, institutions, developers, and deployers, yet professional accountability and final decision authority remain formally concentrated on the individual physician. To address these systemic challenges, we propose a human-centered governance framework —presented as design hypotheses requiring prospective evaluation— encompassing user interface design (embedding risk-adaptive cognitive friction), medical education (establishing targeted, protected AI-free reasoning exercises (“Reasoning Cleanrooms”)), and institutional policies (implementing substantive human oversight and algorithmovigilance). We argue that the goal of digital health governance should not be to maximize automation, but rather to safeguard human cognitive agency, diagnostic vigilance, and layered, distributed accountability within AI-enabled healthcare systems.

## 1 Introduction

Clinical reasoning is a complex cognitive process involving problem representation, hypothesis generation, evidence integration, uncertainty management, and reflective calibration (1). The formation of clinical competence does not rely solely on the retrieval of factual knowledge but depends on the physician’s capacity to construct, update, and calibrate internal mental models during patient encounters (1). Accordingly, the core objective of traditional clinical decision support systems (CDSS) should be to enhance decision accuracy while preserving human cognitive engagement and oversight (2).

Traditional CDSS primarily rely on rule-based logic or discriminative machine learning models (2). Their outputs are typically discrete—such as risk scores, alert flags, or numeric probabilities (e.g., “sepsis risk of 78%”). In these traditional workflows, the division of labor between human and machine is clear: algorithms process raw data, while physicians interpret their clinical significance and construct the overall diagnostic narrative.

However, the rise of generative artificial intelligence (GenAI), and of large language models (LLMs) in particular, is substantially changing this interaction paradigm (3, 4). GenAI systems can synthesize unstructured, complex data from electronic health records (EHRs) into continuous, highly fluent, and action-oriented natural language narratives (3). Rather than merely presenting raw predictive data, GenAI directly synthesizes the diagnostic reasoning process, differential diagnostic analyses, and structured treatment plans on the physician’s behalf (5). Although this may yield efficiency gains in clinical documentation and information retrieval, it may precipitate a qualitative transformation in physician-AI interaction (3, 5). When AI presents a logically coherent clinical narrative at the very entry point of care, it may pre-shape the physician’s initial mental model and diagnostic search space before independent human reasoning has even begun (1, 6). Drawing on cognitive psychology, medical education, and system governance, this paper examines the potential implications of such narrative-based CDSS and proposes a testable, human-centered governance framework.

Because the evidence base on clinician-GenAI interaction is still developing, we adopt explicit epistemic labeling throughout this article: claims grounded in empirical findings are identified as such; mechanisms extrapolated from the automation or cognitive psychology literature are labeled as extrapolations; and our own propositions are framed as hypotheses (“we hypothesize,” “may,” “could”). Recent empirical work underscores the need for this discipline: in a randomized trial, physician access to a large language model did not significantly improve diagnostic reasoning relative to conventional resources, even though the LLM alone outperformed both physician groups (7), and LLMs themselves exhibit human-like cognitive biases (8, 9). Model capability therefore does not translate automatically into improved human-AI collaborative reasoning, and the interaction effects at the center of this Perspective remain open empirical questions.

## 2 The cognitive paradigm shift: from information retrieval to narrative cognitive substitution

Human cognition is naturally endowed with a “cognitive offloading” mechanism, which optimizes working memory capacity by delegating memory or computational tasks to external tools. In routine clinical workflows, using external databases or automated computational tools for specific tasks confers clear cognitive benefits. However, when external systems begin to assume the core responsibilities of synthesizing evidence, generating differential diagnoses, and formulating treatment plans, cognitive offloading may evolve into what we term “cognitive delegation.”

In traditional human-computer interaction research, “automation bias” describes the tendency of humans to uncritically over-rely on automated output under high workload or time pressure (2). We hypothesize that GenAI may amplify this vulnerability through its “narrative fluency” (1, 2). Continuous, grammatically rigorous text that conforms to medical terminology standards creates high “processing fluency,” which may preferentially engage intuitive System 1 processing and reduce the likelihood of rigorous analytic System 2 verification. We treat the System 1/System 2 distinction as a heuristic rather than a settled neurocognitive account: in expert clinicians, System 1 processing largely reflects adaptive expertise—rapid, accurate pattern recognition built through deliberate practice (1)—so the concern raised here is not intuition itself, but the pre-emption of the physician’s own construction of the initial problem representation.

To precisely characterize this distinctive dynamic, we propose Narrative Cognitive Substitution (NCS) as a hypothesized mid-level theoretical construct—a testable configuration hypothesis—rather than an established neurobiological mechanism (1, 5). NCS describes the cognitive restructuring triggered when fluent, action-oriented AI narratives pre-organize diagnostic hypotheses, causal explanations, and management plans, shifting human cognition from active, bottom-up meaning-making to passive, retrospective validation of an exogenously supplied framework.

NCS does not claim that physicians have lost foundational diagnostic skills or blindly accept AI recommendations. Rather, it hypothesizes a change in task structure: confronted with a complete diagnostic narrative, physicians no longer construct reasoning from raw patient data but instead evaluate within a pre-anchored hypothesis space, which may increase the probability of confirmation bias and premature closure (1, 6). Empirical findings in this area are already complex: physician access to an LLM did not significantly improve diagnostic reasoning in a randomized trial (7), and LLMs themselves exhibit human-like cognitive biases (8, 9)—observations consistent with, though not by themselves proof of, the NCS hypothesis.

NCS is not proposed as a novel cognitive architecture. Rather, it is a configuration hypothesis: we propose that in generative environments, established mechanisms—automation bias, anchoring, premature closure, and confirmation bias—are recombined and relocated to an earlier, framing-level stage of the reasoning process (8). Two relatively weighted conceptual distinctions—rather than strict defining boundaries—separate NCS from these constituent mechanisms, since anchors in classical paradigms can also arise from external information, and automation bias can also occur in response to narrative recommendations. First, the relative salience of the provenance and temporal locus of the anchor: in classical anchoring paradigms the anchor typically arises endogenously from the clinician’s own initial impression, whereas in NCS it is characteristically supplied exogenously by the AI before independent reasoning begins. Second, the predominant unit of influence: automation bias has principally been described at discrete decision nodes (accepting or rejecting an alert or a score), whereas NCS is hypothesized to operate on the entire diagnostic narrative, including the hypothesis space, causal chaining, and management framing. These relative distinctions are conceptual, but they yield testable predictions. If NCS occurs, exposure to AI-generated narratives should be associated with contraction of the breadth and diversity of independently generated hypotheses, convergence toward AI-framed formulations, attenuated counterfactual reasoning, reduced detection of deliberately erroneous AI suggestions, and transient performance impairment upon AI withdrawal (Table 1). Three minimum conditions are proposed for identifying NCS empirically: (i) the order of exposure—the AI-generated narrative must be encountered before independent hypothesis generation; (ii) the completeness of the narrative—the output must pre-organize hypotheses, causal explanations, and management plans rather than a single discrete prediction; and (iii) the presence of an independent human-first comparator condition in which the same task is completed without prior AI framing. Without these conditions, observed effects would be difficult to attribute specifically to NCS rather than to overlapping mechanisms. If the expected signatures are absent under controlled conditions meeting these requirements, this would challenge the proposed operationalization or require its refinement. NCS is therefore not proposed as an additional, isolated bias alongside those already catalogued: it is a particular temporal and narrative configuration in which several established mechanisms—automation bias, anchoring, premature closure, and confirmation bias—may interact, with the distinguishing feature being that the framing of the reasoning task itself precedes and shapes independent human reasoning.

**Table 1:** Conceptual distinctions between automation bias, cognitive offloading, and narrative cognitive substitution (NCS).

Construct	Primary output modality	Temporal locus & mechanism	Clinical role & task impact	Primary risks	Empirical signatures & observables
Automation bias	Typically discrete alerts, numeric scores, or binary labels; narrative recommendations are also documented	Post-reasoning validation: uncritical acceptance driven by excessive trust or diminished vigilance; described typically at discrete decision nodes, though narrative outputs may also elicit it (2)	Physician accepts, rejects, or defers to algorithmic recommendations	Omission or misjudgment at a decision point; risk may extend to narrative recommendations	Omission/commission error rates at decision nodes (illustrative) Over-reliance under acute cognitive load
Cognitive offloading	Reference material, computational tools, and broader informational resources, including synthesized content	Task-execution support: strategic redistribution of cognitive load to external resources, extending beyond factual recall to reasoning support	Physician uses external resources to support calculation, memory, or reasoning	Over-reliance on external resources; may extend beyond factual memory	Reduced working memory load Independent hypothesis generation may be preserved, depending on the task and the degree of cognitive delegation
Narrative cognitive substitution (NCS)	Continuous, fluent natural-language clinical narratives and reasoning chains (3, 5)	Pre-emptive framing: textual fluency may engage System 1 processing; construction of the initial reasoning framework is delegated to AI	Physician evaluates and modifies pre-constructed diagnostic and management narratives	Premature closure, anchoring bias, and potential long-term erosion of independent reasoning capacity (1, 6)	Contraction of differential hypothesis breadth Convergence toward AI-framed causal chains Attenuated counterfactual reasoning Impaired performance upon acute AI withdrawal

The distinctions proposed in this table represent a testable conceptual framework rather than empirically validated categories. The empirical signatures listed are candidate outcome measures for future research (Table 2).

## 3 A skill evolution framework for AI-enabled healthcare

Clinical competence is not a static repository of medical knowledge but a dynamic capability built through repeated problem-solving, cognitive friction, and corrective feedback (1). The long-term effects of GenAI on the healthcare workforce cannot be assessed solely by short-term task accuracy; it is necessary to examine the mechanisms of skill acquisition and maintenance under prolonged exposure to narrative-based CDSS (1, 5). We propose a Skill Evolution Framework comprising five trajectories (1, 3, 5):

- Augmentation: GenAI offloads low-level data extraction and synthesis, freeing working memory capacity so that physicians can focus on complex clinical judgment, patient communication, and multimorbidity management (3).
- Restructuring: physicians’ core competence shifts from rote memorization to critical validation, contextual tuning, and ethical oversight of algorithmic output (1).
- Deskilling: owing to long-term reliance on AI-preconstructed narrative frameworks, experienced physicians may experience a gradual decline in independent diagnostic reasoning and hypothesis generation when working without AI assistance (1, 5).
- Never-skilling: because AI systems bypass the cognitive struggle of independent diagnostic synthesis during the critical formative period, trainees and junior physicians may fail to internalize robust clinical mental models (1).
- Mis-skilling: physicians may uncritically internalize latent biases, fabricated causal chains, or non-standard practice patterns embedded in algorithm training sets, integrating them into their personal everyday practice (1, 10).

These five categories are not positions on a single underlying continuum. They differ in the skill process they affect—acquisition (Never-skilling), maintenance (Augmentation, Restructuring, Deskilling), or distortion (Mis-skilling)—and they can co-occur within the same clinician across different tasks: a physician may experience genuine augmentation in documentation-intensive work while becoming progressively dependent on AI framing for complex differential diagnosis. The trajectory a clinician follows is expected to be moderated by baseline expertise, duration and intensity of AI exposure, task complexity, degree of delegation, and the reversibility of change. We therefore present these categories as a conceptual taxonomy rather than empirically validated, mutually exclusive states. It must also be acknowledged that current evidence for deskilling and never-skilling derives largely from conceptual argument, cross-sectional observation, and simulation studies rather than longitudinal demonstrations of clinically meaningful competence loss; recent commentaries and reviews emphasize that this evidence base remains early-stage and heterogeneous (9, 11) (Figure 1).

**Figure 1:**
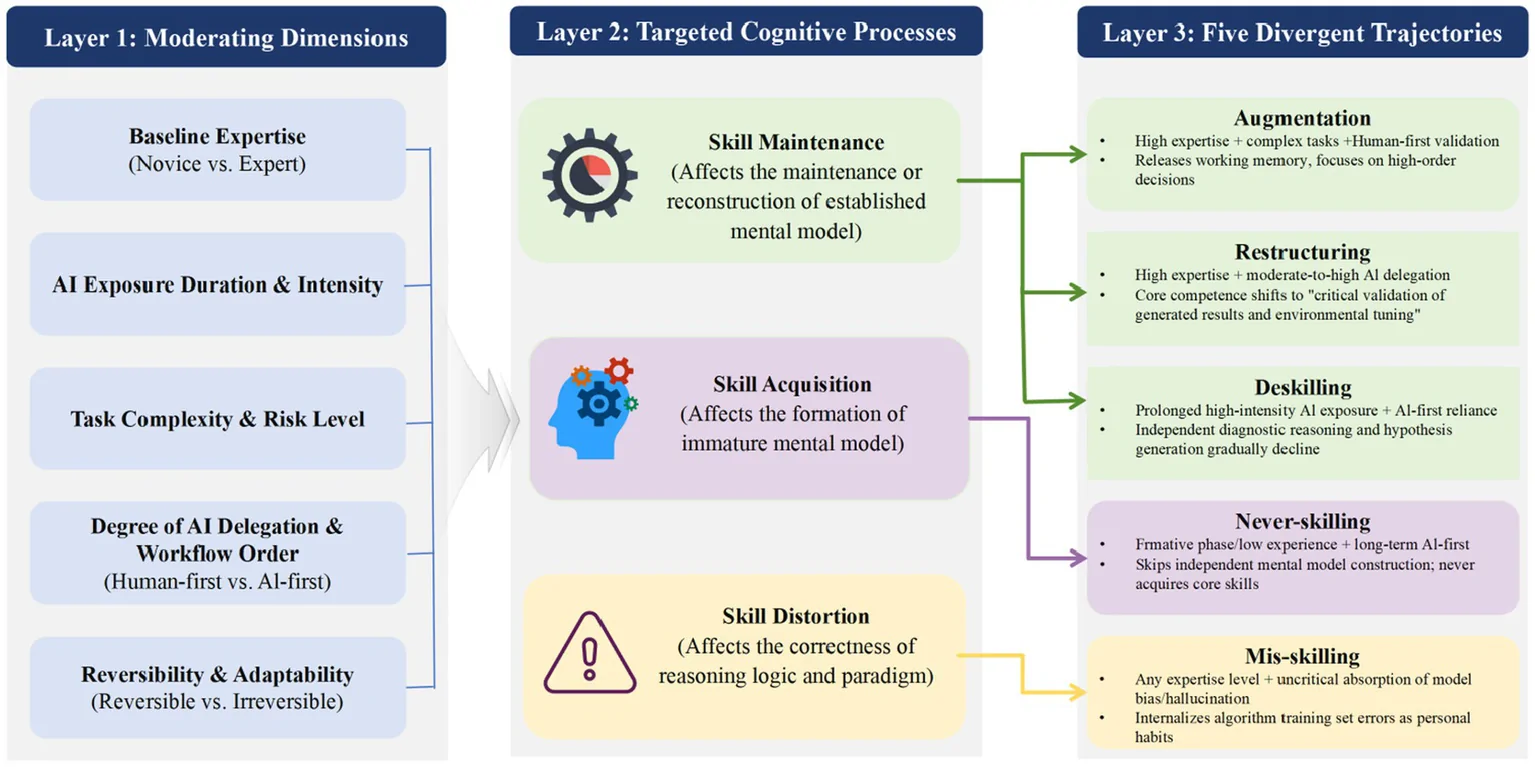
Multi-dimensional taxonomy of skill evolution trajectories under generative AI exposure. Layer 1 (left): moderating dimensions—baseline expertise, duration and intensity of AI exposure, task complexity and risk level, degree of AI delegation and workflow order (human-first vs. AI-first), and reversibility/adaptability. Layer 2 (center): targeted cognitive processes—skill acquisition (formation of immature mental models), skill maintenance (preservation or reconstruction of established mental models), and skill distortion (corruption of reasoning logic). Layer 3 (right): five divergent trajectories—Augmentation, Restructuring, Deskilling, Never-skilling, and Mis-skilling. The same clinician may follow different trajectories across different tasks, and two or more trajectories may coexist. The framework presented in this figure is conceptual and has not been empirically validated; the trajectories and dimensions are proposed for future investigation rather than offered as established categories.

To safeguard the healthy development of the healthcare talent pipeline, it is important to recognize the potential influence of workflow order on human-AI synergy: we hypothesize that when humans think independently first and then consult AI for validation (human-first), independent cognitive capacity may be better preserved—a context-dependent possibility requiring empirical testing rather than an established effect; when AI generates the framework directly (AI-first), practice may, over time, drift toward erosion of clinical skill. The relevant trajectory is expected to differ by population and should be kept conceptually distinct: for experienced physicians the plausible concern is primarily Deskilling, that is, deterioration of previously acquired competence; for trainees in the formative period the corresponding concern is Never-skilling, that is, incomplete initial acquisition. In general clinical practice, the two processes should therefore be evaluated separately rather than combined under a single label (2, 6).

## 4 Distributed responsibility and the accountability gap

Legal and ethical accountability for AI-supported healthcare is not governed by a single universal allocation rule: it depends on the jurisdiction, the regulatory classification and intended use of the system, its deployment modality, applicable negligence and product-liability standards, contractual arrangements, and the causal contribution of the actors involved (12–14). Once GenAI is embedded in the care pathway, decision influence is distributed among software developers, providers, deployers, hospital administrators, and clinical end-users (12, 13). Much current use of general-purpose GenAI also occupies a regulatory grey zone, in which tools without medical-device certification become action-guiding through gradual workflow drift rather than deliberate deployment decisions (12).

For analytical clarity, we distinguish five related but non-equivalent layers of responsibility: professional accountability (clinicians’ duties of competence, verification, and documentation); ethical responsibility (transparency toward patients and colleagues about AI involvement); organizational responsibility (institutions’ duties in selecting, configuring, resourcing, and supervising deployed systems); regulatory obligations (compliance duties of providers and deployers under instruments such as the EU AI Act and the Medical Device Regulation); and legal liability (the ex post allocation of fault under jurisdiction-specific law) (12–15). We likewise distinguish human control (the immediate ability to intervene on a specific output), human oversight (the systemic arrangements by which humans govern AI use), and final decision authority (formal responsibility for the clinical decision); regulatory and medico-legal frameworks treat these constructs differently, and conflating them invites category errors (12–14).

Within this layered landscape, we highlight three structural fault lines that may arise when narrative-based CDSS becomes deeply embedded in care pathways (16, 17):

- Transparency gap: the opacity of black-box architectures and the non-deterministic generative characteristics of LLMs make it difficult for physicians to trace the clinical basis of a given narrative recommendation. We note that the inability to inspect model parameter weights is not equivalent to the absence of clinically useful transparency: what matters for clinical oversight is whether the system provides clinical provenance, traceability of the evidence supporting each recommendation, an explanation of the basis on which recommendations were generated, and documentation of model limitations and intended use. The gap addressed here therefore concerns the absence or inadequacy of these oversight-relevant elements rather than access to underlying parameters (10, 16).
- Control gap: although physicians formally retain the final decision-making authority (override power)—the allocation of that authority depends on the jurisdiction, workflow, institutional arrangements, and regulatory classification of the system—pre-constructed narratives may narrow the actual search space, weakening physicians’ genuine cognitive control over the initial decision framework (2, 6).
- Attribution gap: when adverse clinical events occur, allocating responsibility among algorithmic defects (developers), deployment protocols (healthcare institutions), and supervisory practices (physicians) becomes extremely complex and jurisdiction-dependent (12, 16, 17).

This misalignment gives rise to what we term a responsibility paradox: professional accountability and final decision authority remain formally concentrated on the individual physician, while the cognitive inputs to that decision are shaped by systems the physician did not design, cannot fully inspect, and cannot unilaterally govern (12, 17). Notably, the EU AI Act’s human-oversight framework (Regulation (EU) 2024/1689, Article 14) does not simply relocate responsibility to individual clinicians: for high-risk AI systems within the scope of the Act, it obliges providers to design systems that enable effective oversight, and deployers to allocate competent, authorized personnel and adequate resources to oversight functions (13, 14). This oversight regime does not, however, apply uniformly to every form of generative AI used in clinical workflows. These descriptive labels do not themselves determine a system’s regulatory status, which depends on its classification, intended purpose, and deployment context. We therefore distinguish three analytically distinct but potentially overlapping dimensions and their respective allocative logics: (i) high-risk AI systems regulated under the Act, to which the Article 14 human-oversight requirements and the associated provider and deployer obligations apply; (ii) general-purpose or non-medical-device systems used in clinical workflows, which may fall outside dedicated medical-device oversight and whose applicability under the Act depends on the system’s classification, intended purpose, and mode of deployment; and (iii) the national professional, negligence, organizational, and product-liability rules that continue to govern clinical practice irrespective of the regulatory status of the system. Provider duties under Article 14, deployer duties concerning the allocation of competent human oversight, and national professional and liability rules are distinct obligations that should not be conflated. If decision influence is distributed, we propose—as an analytical and normative framework rather than a description of how liability is already allocated in every jurisdiction—that accountability should be distributed and layered accordingly (12, 15). We emphasize that the legal analysis in this article is conceptual—intended to identify structural gaps rather than to provide jurisdiction-specific legal interpretation.

## 5 A human-centered multi-level governance framework

To effectively mitigate the hypothesized cognitive risks of NCS while harnessing the operational benefits of GenAI, digital health governance must move beyond back-end model accuracy validation and extend comprehensively to front-end cognitive safety and institutional policy (18). We propose a human-centered governance framework with three dimensions. The interventions below should be read as design hypotheses—plausible governance mechanisms whose effectiveness, and whose potential unintended consequences, require prospective empirical testing rather than being assumed.

### 5.1 Interface governance: risk-adaptive cognitive friction

The human-computer interaction (HCI) design of clinical GenAI should proactively avoid seamless, frictionless hyper-automation (2, 19). To counteract the System 1 heuristic processing that may be induced by textual fluency, interfaces should embed cognitive friction—design elements that may promote System 2 analytic evaluation at critical decision points (10, 16):

- Evidence mapping: explicitly link each conclusion in the narrative to the original EHR data or authoritative clinical guidelines (10).
- Uncertainty visualization: clearly indicate missing data, calibrated uncertainty where the system provides it, the provenance and evidence strength of key claims, and explicit statements of model limitations, alongside ambiguity in the differential diagnosis (10).
- Mandatory human control points: requiring physicians to record an independent initial diagnostic impression before AI recommendations are displayed may help preserve autonomous reasoning; however, forced-entry designs of this kind may also reinforce the clinician’s initial anchoring, invite superficial compliance, and add workflow burden. Whether this form of cognitive friction improves decisions is likely to depend on expertise, task complexity, clinical risk, time pressure, and interface design, and should be evaluated rather than assumed (16).

Cognitive friction should also be risk-adaptive, and we refine our earlier position on time-critical settings. Urgency and risk do not necessarily move in opposite directions: rapidly evolving, high-stakes situations may warrant strong safeguards despite severe time constraints. The design goal in emergency settings is therefore not less protection but low-interruption, high-salience safeguards—prominent uncertainty and provenance indicators, and mandatory double-checks for the highest-risk actions—rather than lengthy friction-inducing workflows. In routine diagnosis and complex ward rounds, more deliberate cognitive friction can be applied to safeguard analytic reasoning.

### 5.2 Educational governance: protecting independent mental models

Medical education must be reformed to ensure that students establish robust internal clinical mental models before relying on GenAI assistants for clinical reasoning (1). We propose establishing “Reasoning Cleanrooms” as protected AI-free reasoning exercises: specifically, AI-free formative exercises, selected rotations and simulation sessions, and defined competency assessments during which trainees must complete the full process “from raw data to hypothesis formation” without AI assistance. We do not propose a blanket prohibition of AI tools across broad phases of training—a far stronger intervention that would require separate educational justification; the aim is scheduled protection of the reasoning process, not isolation from technology (1). Staged human-AI collaborative training should introduce courses on AI narrative review, hallucination identification, and algorithmic bias auditing in intermediate and advanced training (1, 5). Regular “AI-off practice”—offline diagnostic drills for practicing physicians—may help assess and preserve clinical competence in the event of system outages or extreme edge cases (1).

### 5.3 Institutional governance: implementing substantive human oversight

Healthcare institutions must establish policies ensuring that human oversight of high-risk clinical AI is “substantive” rather than “perfunctory” (17, 18, 20). End-to-end audit logging of AI outputs, model versions, physician modifications, and final orders can support post-deployment surveillance and accountability, consistent with emerging algorithmovigilance frameworks (15). Two caveats deserve emphasis. First, interaction duration is not a valid proxy for substantive oversight: a long session may reflect rubber-stamping, whereas effective review may be rapid. Second, extensive monitoring itself carries risks—clinician surveillance, privacy concerns, workload, and defensive practice—that must be weighed and explicitly governed (15).

## 6 From individual cognition to system-level consequences

If the mechanisms hypothesized here operate at scale, their consequences would extend well beyond individual physicians to the public health capacities of health systems. First, workforce competence: cohorts trained and practicing in AI-first environments may develop differently distributed competences, with implications for workforce planning and institutional resilience (11). Second, diagnostic variation: if large numbers of clinicians reason within AI-generated frames, diagnostic practice may become pseudo-homogenized—superficially more consistent while inheriting the systematic biases and blind spots of the underlying models (8, 9). Third, equity: because LLMs can encode and amplify biases against underrepresented populations (8), narrative-level influence may propagate disparities in diagnostic thoroughness and, ultimately, patient safety. Fourth, system resilience: institutions whose workflows presuppose AI support must retain the capacity to deliver safe care during model failures, outages, or distribution shifts—a dimension increasingly emphasized in post-deployment governance frameworks (13, 15). Framed this way, cognitive agency is not merely a matter of individual physician discipline; it is a population-level asset and a precondition for safe digital public health infrastructure. These system-level consequences remain prospective hypotheses; each requires dedicated empirical investigation before it can be treated as an established effect.

## 7 A research agenda for empirical validation

The propositions advanced in this Perspective are, in the main, hypotheses. We therefore outline a research agenda organized around measurable outcomes (Table 2). Priority questions include: whether independent hypothesis generation contracts following sustained AI exposure; whether differential breadth and calibration differ under AI-first versus human-first workflows; whether clinicians detect intentionally erroneous AI suggestions; how performance changes after temporary AI withdrawal; and whether cognitive-friction interfaces and Reasoning Cleanrooms improve downstream patient-safety outcomes without imposing unsustainable cognitive or workflow costs. Answering these questions will require collaboration among clinical reasoning researchers, human-factors specialists, educators, and governance scholars, and would connect the present framework to the empirical literature on clinician-LLM interaction (7–9, 11).

**Table 2:** A research agenda for empirically testing NCS, skill evolution trajectories, and governance safeguards.

Research question	Candidate outcome measures	Illustrative designs
Does AI-first framing narrow independent reasoning?	Number and diversity of independently generated hypotheses; breadth of differential diagnosis; convergence to AI-framed hypotheses	Randomized vignette experiments comparing AI-first and human-first workflows
Is NCS distinguishable from automation bias and anchoring?	Detection rate of deliberately seeded AI errors; frequency of counterfactual reasoning; anchoring indices	Controlled experiments embedding erroneous AI narratives; bias-disambiguation paradigms
Does dependence develop and persist over time?	Performance after temporary AI withdrawal; longitudinal skill retention; confidence calibration	Longitudinal cohort studies; withdrawal trials; simulation-based assessment
Do proposed safeguards work as intended?	Quality versus superficiality of compliance; cognitive workload; override rates; patient-safety outcomes	Prospective implementation studies of cognitive-friction interfaces, Reasoning Cleanrooms, and audit mechanisms
What are the system-level consequences?	Diagnostic variation across institutions; subgroup safety disparities; care quality during AI outages	Health-services research; natural experiments during outages; algorithmovigilance surveillance (15)

## 8 Conclusion

Generative AI is a milestone in the evolution of clinical decision support, but narrative-based output also introduces cognitive challenges that remain to be fully characterized. By creating high processing fluency, GenAI may trigger narrative cognitive substitution and reshape the mechanisms through which clinical reasoning occurs. The ultimate aspiration of digital health governance should not be the pursuit of full automation, but rather the sustained preservation of human cognitive agency, diagnostic vigilance, and layered, distributed accountability in the age of AI assistance.

## Data Availability

The original contributions presented in the study are included in the article/supplementary material, further inquiries can be directed to the corresponding authors.
